# Association between red blood cell distribution width and ischemic stroke recurrence in patients with acute ischemic stroke: a 10-years retrospective cohort analysis

**DOI:** 10.18632/aging.204657

**Published:** 2023-04-12

**Authors:** Zhan Shen, Ying Huang, Ying Zhou, Jingying Jia, Xian Zhang, Tingting Shen, Shengjie Li, Siyang Wang, Yunxiao Song, Jie Cheng

**Affiliations:** 1 Department of Geratology, Shanghai Xuhui Central Hospital, Fudan University, Shanghai, People’s Republic of China; 2 Department of Neurology, Shanghai Xuhui Central Hospital, Fudan University, Shanghai, People’s Republic of China; 3 Department of General Medicine, Shanghai Xuhui Central Hospital, Fudan University, Shanghai, People’s Republic of China; 4 Department of Central Laboratory, Shanghai Xuhui Central Hospital, Fudan University, Shanghai, People’s Republic of China; 5 Shanghai Internet Hospital Engineering Technology Research Center, Shanghai Xuhui Central Hospital, Fudan University, Shanghai, People’s Republic of China; 6 Department of Clinical Laboratory, Shanghai Xuhui Central Hospital, Fudan University, Shanghai, People’s Republic of China; 7 Department of Urinary Surgery, Shanghai Xuhui Central Hospital, Fudan University, Shanghai, People’s Republic of China

**Keywords:** stroke, ischemic stroke, red blood cell distribution width, recurrence, cohort

## Abstract

Numerous studies have reported that a higher red blood cell distribution width (RDW) level was associated with adverse outcomes in patients with the first stroke. However, no studies have examined the association between RDW and recurrent ischemic stroke. We performed a population-based cohort data analysis from 2007 to 2017. Baseline RDW was measured in 6402 first ischemic stroke participants, who were followed for about five years on average. During 62 months of median follow-up, 205 participants (3.20%) reported a recurrence (self-reported). RDW showed a nonlinear relationship with the risk of ischemic stroke recurrence. When RDW was assessed as quartiles (quartile 1, RDW<12.4; quartile 2, 12.4 to 12.8; quartile 3,12.8 to 13.3, quartile4, RDW>13.3), compared with the reference group (quartile 1), the hazard ratios (HRs) of ischemic stroke recurrence were 1.372 (95% confidence interval [CI]=0.671-2.805, P=0.386) in quartile 2, 1.835 (95% CI=1.222-2.755, P=0.003) in quartile 3, and 1.732 (95% CI=1.114-2.561, P<0.001) in quartile 4. The trend test was significant (P<0.001). When quartiles 3 and 4 were combined, the adjusted HR of ischemic stroke recurrence was 1.439 (95% CI=1.330-1.556, P<0.001) compared with the combined quartiles 1 and 2 subgroups. This study demonstrated that elevated RDW levels were positively associated with an increased risk of recurrent ischemic stroke. RDW can provide a new perspective for initial risk assessment and identify high-risk patients early. Further research is required to confirm our results.

## INTRODUCTION

In 2019, stroke was the second-leading cause of death among adults around the world [1]. The global prevalence of ischemic strokes has reached 76.3 million [1], and an incidence of stroke occurs once every 5 seconds worldwide [2]. East Asia, Southern Sub-Saharan Africa, Eastern Sub-Saharan Africa, and Southeast Asia had the most significant load of ischemic stroke between 1990 and 2019. In particular, China (EAPC 1.10, 95% CI 1.00-1.20) had the most pronounced increases in the age-standardized incidence rate of ischemic stroke. Globally, there was an increase in ischemic stroke incidence with increasing age, especially in women 50 to 69 years of age or older [3].

Having had a first ischemic stroke, there is an increased risk of recurrence [1]. According to the study, there were 1.9 ischemic stroke recurrences per 100 patient-years, while 2.19 ischemic stroke deaths per 100 patient-years [4]. Therefore, it is more likely that stroke prevention can be achieved if patients at increased risk of recurrence are identified early so that special attention and resources can be devoted to them. To accomplish this, precise risk prediction models are required. It has been attempted to identify predictors of recurrent ischemic strokes in many studies [5–9]. The Essen Stroke Risk Score (ESRS) [8] and the modified ESRS [7] are the most well-known scores to evaluate the long-term (1 -year) risk of ischemic stroke recurrence. Patient age, several comorbidities (such as hypertension, diabetes, etc.), a history of myocardial infarction, and smoking status were included to form ESRS. In addition, waist circumference, stroke subtype by etiology, and sex were selected to form a modified version. The ESRS and the modified ESRS were introduced in 2006 and 2013. Since then, developments in the diagnosis and therapy of stroke have improved. Thus, other indices independent of traditional ESRS and modified ESRS indices could characterize high-risk recurrent patients.

Recently, numerous studies have reported that a higher level of RDW was associated with adverse outcomes in patients with the first stroke [10–14]. For example, João Pinho et al. [11] performed a single-center retrospective cohort study and found that patients in the higher quartiles of RDW presented a lower 1-year survival (p < 0.001). Furthermore, Jostein Lappegård et al. [12] reported that subjects with RDW above the 95 percentile had a 55% higher risk of stroke than those in the lowest quintile (HR: 1.55, 95% CI: 1.16-2.06).

However, no studies have examined the prospective association between RDW and recurrent ischemic stroke. Thus, we hypothesized that high levels of RDW increase the risk of ischemic stroke recurrence. A population-based cohort analysis was conducted to address this research gap to examine the association between RDW at baseline and recurrent ischemic stroke over time.

## RESULTS

### Baseline characteristics

Of the 6402 ischemic stroke participants, 205 suffered ischemic stroke recurrence. The baseline characteristics are shown in Table 1 according to the first ischemic stroke case and recurrence. The median follow-up period was 62 months (range, 1–115 months), and 578 (8.28%) patients were excluded during the follow-up period. The median level of RDW was 12.8% (range, 10.8%–25.2%). The mean level of RDW in the first stroke population was 12.87%, which was significantly lower (P<0.001) than that of the recurrence of the ischemic stroke group (RDW: 13.44%).

**Table 1:** Baseline characteristics of all participants by ischemic stroke and recurrent.

**Variables**	**All**	**Ischemic stroke**	**Recurrent**	**P value**
Number, n	6402	6197	205	
Age (y)	74.41±11.20	74.41±11.79	74.36±11.72	0.943
Male/Female	3259/3143	3155/3042	104/101	0.960
Hypertension (Yes/No)	3074/3328	2981/3216	93/112	0.440
Diabetes mellitus (Yes/No)	1422/4980	1373/4824	49/156	0.554
Smoking (Yes/No)	383/6019	370/5827	13/192	0.826
Drinking (Yes/No)	205/6197	198/5999	7/198	0.861
BMI (Kg/m^2^)	22.74±2.89	22.74±2.89	22.73±2.93	0.972
CHD (Yes/No)	429/5973	416/5781	13/192	0.834
mRS (≥3/<3/missing)	3908/1836/658	3776/1781/640	132/55/18	0.447
NIHSS, median (IQR)	6 (3-18)	6 (3-17)	7 (3-18)	0.156
NIHSS, n (missing)	598	572	26	-
WBC (10^9^/l)	6.16±2.29	6.15±2.31	6.18±1.51	0.850
RBC (10^12^/l)	4.08±0.60	4.08±0.60	4.13±0.62	0.194
HGB (g/l)	124.81±18.07	124.78±18.03	125.45±19.06	0.589
PLT (10^9^/l)	198.21±62.20	198.15±61.24	199.86±84.81	0.687
AST (u/l)	21.26±9.63	21.25±9.65	21.41±9.07	0.809
ALT (u/l)	19.82±14.99	19.80±15.07	20.17±12.58	0.722
GGT (u/l)	28.38±32.91	28.27±32.23	31.41±48.08	0.160
BUN (mmol/l)	5.86±1.95	5.86±1.97	5.79±1.51	0.586
CREA (umol/l)	76.74±41.71	76.69±42.16	78.16±26.23	0.607
GLU (mmol/l)	6.15±1.90	6.14±1.92	6.36±1.45	0.028
RDW (%)	12.89±0.86	12.87±0.82	13.44±1.54	<0.001

BMI, body mass index; CHD, coronary heart disease; WBC, white blood cell count; BUN, urea nitrogen; CREA, creatinine; GLU, glucose; RBC, red blood cell count; HGB, hemoglobin; AST, aspartate aminotransferase; ALT, alanine aminotransferase; GGT, gamma-glutamyl transpeptidase.

The patients were divided into four groups based on the quartiles of RDW. Compared with patients showing low RDW (quartile 1–2), those with a high RDW (quartile 3–4) were significantly older (P <0.001) and had higher BMI values (P=0.011) (Table 2). In addition, the levels of white blood cell count (WBC), urea nitrogen (BUN), creatinine (CREA), and glucose (GLU) were significantly higher (P<0.05) in the high RDW (quartile 3–4) group than low RDW (quartile 1–2) group (Table 2). However, the levels of red blood cell count (RBC) and hemoglobin (HGB) were significantly lower (P<0.05) in the high RDW (quartile 3–4) group than the low RDW (quartile 1–2) group (Table 2).

**Table 2:** Comparison of baseline characteristics, stratified according to median baseline RDW level.

	**RDW (Q1-Q2)**	**RDW (Q3-Q4)**	**P value**
Number, n	3475	2927	
Age (y)	72.74±10.95	76.40±11.15	<0.001
Male/Female	1750/1725	1509/1418	0.341
Hypertension (Yes/No)	1666/1809	1408/1519	0.898
Diabetes mellitus (Yes/No)	767/2708	655/2272	0.769
Smoking (Yes/No)	209/3266	174/2753	0.907
Drinking (Yes/No)	109/3366	96/2831	0.746
BMI (Kg/m^2^)	22.66±2.88	22.84±2.90	0.011
CHD (Yes/No)	241/3234	188/2744	0.404
mRS (≥3/<3/missing)	2010/966/499	1898/870/159	0.403
NIHSS, median (IQR)	6 (3-18)	6(3-18)	0.457
NIHSS, n (missing)	312	286	-
WBC (10^9^/l)	6.05±2.11	6.28±2.47	<0.001
RBC (10^12^/l)	4.11±0.54	4.04±0.67	<0.001
HGB (g/l)	127.28±16.27	121.85±19.60	<0.001
PLT (10^9^/l)	198.92±58.10	197.38±66.76	0.323
AST (u/l)	21.22±8.63	21.30±10.70	0.763
ALT (u/l)	19.70±12.53	19.95±17.48	0.496
GGT (u/l)	28.42±30.84	28.33±35.23	0.913
BUN (mmol/l)	5.78±1.77	5.95±2.14	<0.001
CREA (umol/l)	74.11±28.97	79.89±52.89	<0.001
GLU (mmol/l)	6.10±1.92	6.20±1.87	0.024
RDW (%)	12.35±0.34	13.53±0.85	<0.001

BMI, body mass index; CHD, coronary heart disease; Q, quartile; WBC, white blood cell count; BUN, urea nitrogen; CREA, creatinine; GLU, glucose; RBC, red blood cell count; HGB, hemoglobin; AST, aspartate aminotransferase; ALT, alanine aminotransferase; GGT, gamma-glutamyl transpeptidase.

### Association between RDW and the recurrence of ischemic stroke

**Figure 1:**
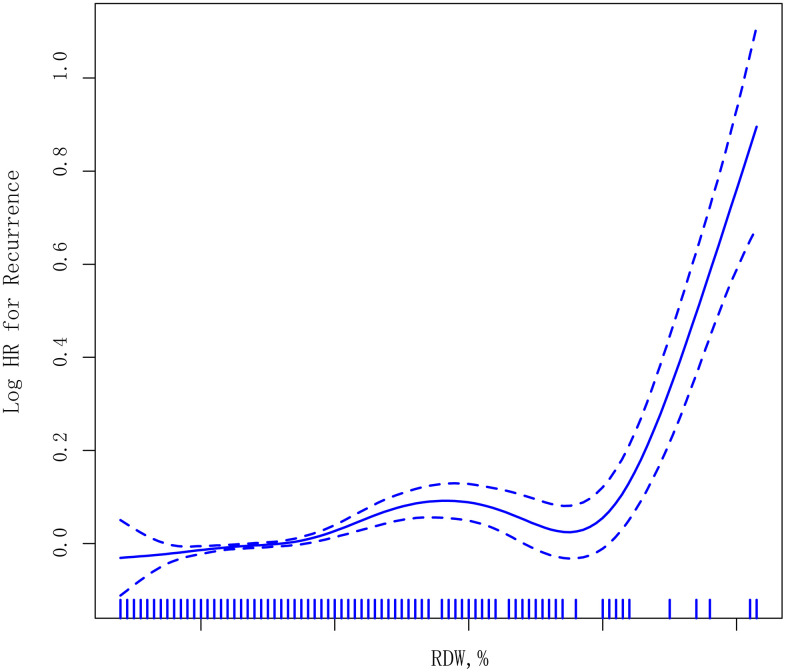
**Smoothing curves derived from generalized additive models illustrating the association between RDW levels (as a continuous variable) and recurrent ischemic stroke.** The median follow-up period was 62 months (1–115 months).

A GAM model was used to identify the relationship between the risk of ischemic stroke recurrence and baseline RDW. Figure 1 shows a nonlinear smoothing curve (solid line) and 95% CI (dashed lines) between RDW and the HR of stroke recurrence, with adjustments for covariables.

Consistently, when RDW was assessed as quartiles, compared with the reference group (quartile 1, RDW ≤12.4%), the HRs of recurrence of ischemic stroke was 1.372 (95% CI=0.671–2.805, P=0.386) in quartile 2 (12.8%≥RDW>12.4%), 1.835 (95% CI=1.222–2.755, P=0.003) in quartile 3 (13.3%≥RDW>12.8%), and 1.732 (95% CI=1.114–2.561, P<0.001) in quartile 4 (RDW>13.3%), with a significant trend test (P<0.001) after adjusting for covariables. When quartiles 3 and 4 were combined, the adjusted HR of recurrence of ischemic stroke was 1.439 (95% CI=1.330–1.556, P<0.001) compared with the combined quartiles 1 and 2 subgroups (Table 3).

**Table 3:** Association of baseline RDW levels with recurrent ischemic stroke.

	**Crude model**		Adjusted model*	
	**HR (95%CI)**	**P value**	**HR (95%CI)**	**P value**
Age	1.450 (1.152-1.845)	<0.001	1.221 (1.01-1.51)	0.012
Sex	1.006 (0.765-1.323)	0.966	1.015 (0.77-1.34)	0.919
BMI	0.999 (0.952-1.048)	0.970	1.001 (0.95-1.05)	0.986
Smoking	1.267 (1.108-1.671)	0.022	1.121 (1.02-1.31)	0.046
Drinking	1.067 (0.502-2.267)	0.867	1.073 (0.41-2.82)	0.887
Diabetes mellitus	1.100 (0.798-1.516)	0.561	1.170 (0.81-1.70)	0.410
Hypertension	0.897 (0.681-1.181)	0.439	0.864 (0.64-1.17)	0.344
CHD	0.942 (0.537-1.652)	0.836	0.924 (0.51-1.68)	0.795
mRS (≥3/<3)	1.035 (0.794-1.349)	0.801	1.015 (0.903-1.118)	0.829
NIHSS	1.001 (0.961-1.043)	0.947	1.002 (0.961-1.045)	0.919
WBC	1.015 (0.954-1.079)	0.641	1.001 (0.937-1.070)	0.973
RBC	1.151 (0.902-1.470)	0.258	1.414 (0.768-2.650)	0.266
HGB	1.003 (0.995-1.011)	0.508	0.994 (0.974-1.015)	0.568
PLT	1.001 (0.999-1.004)	0.206	1.001 (0.999-1.004)	0.229
AST	1.004 (0.992-1.016)	0.552	1.004 (0.979-1.029)	0.772
ALT	1.002 (0.994-1.010)	0.638	0.998 (0.982-1.014)	0.800
GGT	1.003 (1.000-1.006)	0.086	1.002 (0.998-1.006)	0.262
BUN	0.961 (0.885-1.044)	0.344	0.919 (0.832-1.014)	0.091
CREA	1.001 (0.998-1.003)	0.666	1.003 (0.999-1.006)	0.159
GLU	1.051 (0.989-1.117)	0.109	1.056 (0.992-1.123)	0.087
RDW				
Quartile				
Q1 (<12.4)	Ref		Ref	
Q2 (12.4 to 12.8)	1.472 (0.730-2.966)	0.280	1.372 (0.671-2.805)	0.386
Q3 (12.8 to 13.3)	1.773 (1.192-2.638)	0.005	1.835 (1.222-2.755)	0.003
Q4 (>13.3)	1.724 (1.118-2.238)	<0.001	1.732 (1.114-2.561)	<0.001
P for trend		<0.001		<0.001
Categories				
Q1-Q2 (<12.8)	Ref		Ref	
Q3-Q4 (>12.8)	1.442 (1.341-1.551)	<0.001	1.439 (1.330-1.556)	<0.001

*Adjusted for age, sex, hypertension, diabetes mellitus, smoking, drinking, body mass index, and coronary heart disease. Q, quartile.

**Figure 2:**
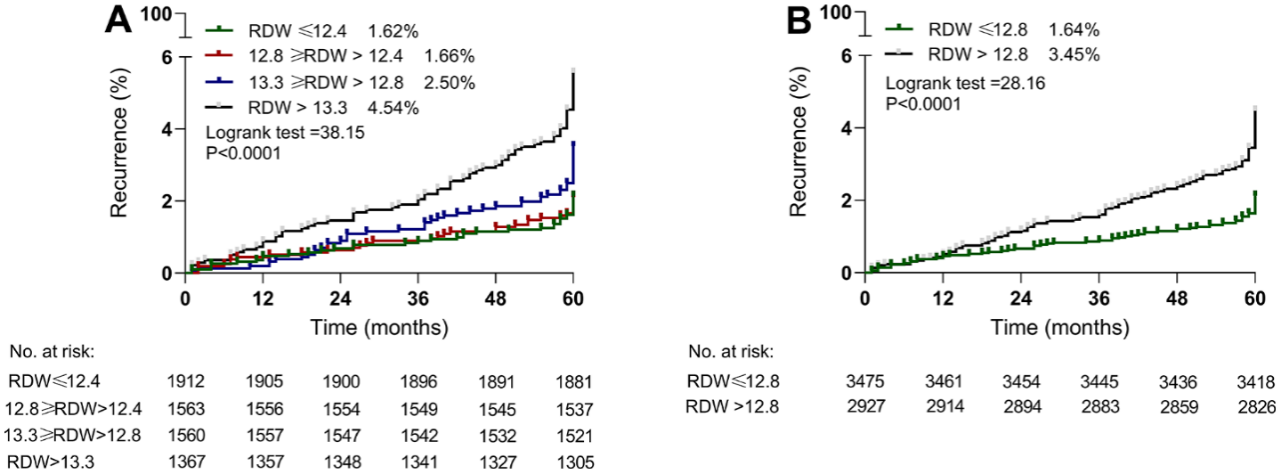
**Kaplan–Meier estimates of recurrent ischemic stroke according to baseline RDW levels.** (**A**) RDW was assessed as a categorical variable based on quartile: quartile 1, RDW≤12.4%; quartile 2, 12.8%≥RDW>12.4%; quartile 3, 13.3%≥RDW>12.8%; quartile 4, RDW>13.3%. (**B**) Quartiles 1 and 2 were combined into one group, and quartiles 3 and 4 were combined into one group.

The impact of baseline RDW levels on ischemic stroke recurrence was analyzed using Kaplan–Meier analysis (Figure 2). Participants with RDW (Q4) in the highest quartile had a high rate of recurrence events (4.54% vs. 2.50% vs. 1.66% vs. 1.62%, P<0.0001, Figure 2A) compared with those with RDW in the higher quartile (Q2), followed by the lower quartile (Q3) and then the lowest quartile (Q1). When quartiles 3 and 4 were further combined, the rate of recurrence events was 3.45% (Figure 2B, P<0.0001) compared with quartiles 1 and 2 (1.64%).

Furthermore, our results also found that risk factors for recurrent stroke were age (HR, 1.221 [95% CI, 1.01-1.51; P=0.012] and smoking (HR, 1.121 [95% CI, 1.02-1.31]; P=0.046) (Table 3).

### Sensitivity analysis

Sensitivity analysis was performed to assess if the results would change when lost follow-up patients (n=175) were included. First, all the lost follow-up patients (n=175) were considered stroke recurrence. Similar results (Supplementary Table 1) were observed with the above results (Table 3). Second, all the lost follow-up patients (n=175) were considered as non-stroke recurrence. Similar results (Supplementary Table 1) were observed with the above results (Table 3).

## DISCUSSION

The RDW is calculated by automated equipment during a complete blood count to reflect variations in the size of circulating red cells (anisocytosis) [15]. Despite mounting evidence that RDW increases the risk of cardiovascular and cerebrovascular disease, including stroke, until now, there has been no definitive link between RDW and recurrent stroke. This study is the first to report the prospective association between baseline RDW levels and recurrent ischemic stroke in a large-sample, population-based cohort study. We found that higher baseline RDW levels were graded and independently associated with a higher risk of recurrent stroke. Furthermore, the association between higher RDW levels and recurrent ischemic stroke was attenuated but not eliminated by adjusting for multiple potential confounders.

The results of this study in patients with a previous stroke whose treatment followed international guideline recommendations showed a rate of recurrent ischemic stroke events of 3.20%. The rate is consistent with previous results from randomized controlled trials: 2.83% in the ENGAGE AF-TIMI 48 [16]; 2.32% in RELY [17]; and 2.79% in ROCKET AF [18].

Increased RDW levels and adverse cerebrovascular outcomes have received considerable attention in various patient populations. A comprehensive meta-analysis of 31 studies with 3,487,896 patients found that elevated RDW was a risk factor in ischemic stroke (OR/RR 1.528; 95% CI = 1.372-1.703) [19]. A previous analysis of 25,992 subjects participating in the fourth survey of the Tromsø Study found that a 1% increment in RDW yielded a 13% higher risk of stroke [12]. In addition, one retrospective cohort study showed that the risk of 1-year mortality and shorter survival was higher in patients with RDW values over 14.5% [20].

In the present study, higher levels of RDW were associated with an increased risk of recurrent ischemic stroke, even after adjustment for confounding factors. He et al. [21] reported that higher mean RDW level from prior intravenous thrombolysis to 72 h after intravenous thrombolysis was associated with an increased risk of hemorrhagic transformation and recurrent stroke. Furthermore, Xue et al. [22] reported that RDW is related to stroke severity and adverse functional outcomes in individuals with ischemic stroke at three months. RDW and serum levels of neuron-specific enolase had a strong correlation (r=0.275, 95% CI: 0.187-0.359, P<0.001), which indicated a substantial positive relationship between RDW and neuronal damage in individuals with acute ischemic stroke [23]. Our study confirms and extends these findings that higher baseline RDW is associated with an increased risk of recurrent ischemic stroke.

The underlying mechanisms of RDW are not examined in this study, but previous studies suggest that inflammation and oxidative stress may play an important role in ischemic stroke [24]. As a result of inflammation, red blood cells have a lower survival rate, and erythropoietin production is inhibited, leading to a high level of RDW [25]. An imbalance of oxidative stress will damage nucleic acids, proteins, and lipids, affecting the survival of red blood cells. Consequently, the red blood cell membrane may be damaged [26], red blood cells may become more fragile, maturation and longevity may be reduced, and RDW may rise [27]. The cascade of cerebral ischemic injury involves oxidative stress and inflammatory activity [28]. Thus, we speculate that an imbalance in oxidative stress and an underlying inflammatory state may be reflected in higher levels of RDW, which is associated with adverse clinical outcomes. Future studies will be required to confirm this hypothesis, and further research is required to investigate the determinants of RDW in populations with recurrent ischemic stroke.

Hyperglycemia is frequent in acute ischemic stroke and is linked to a greater ultimate infarct volume and a poorer clinical prognosis [29, 30]. Compared to individuals with normal fasting glucose levels, those with impaired fasting glucose had a poorer functional outcome (OR=2.77; 95% CI=1.54-4.97) [30]. In this study, we also found that the levels of GLU were significantly higher (P=0.028) in the recurrent group than first stroke group. The Cox regression analysis showed that a high level of GLU was a risk factor for recurrent stroke (HR=1.056, P=0.087). Moreover, Peng et al. [31] found that both low and high levels of BUN were associated with higher risks of total and ischemic stroke. However, in the present study, both low and high levels of BUN were not associated with an increased risk of recurrent ischemic stroke.

The strengths of our study include its relatively large sample size and all assays being conducted in one laboratory. In addition, all RDW measurements were performed the same way. Our analyses are based on real-life experiences and may contribute to reducing the incidence of recurrent stroke by monitoring RDW levels.

In this study, several limitations must be highlighted. First, the results were not externally validated since all data came from the same source. In other populations, more studies are needed to confirm the association between RDW and recurrent stroke. Second, although oral anticoagulation followed international guideline recommendations, pharmacological treatments other than oral anticoagulation were not investigated. In addition, the dose of oral anticoagulation may be modified based on the situation of the disease. Third, during the follow-up period, 578 (8.28%) patients (voluntary withdrawal=95, missing data=171, lost to follow-up=175, surgical treatment=63, death but not owing to stroke recurrence =74) were excluded from the study. The reason for 403 patients dropping out of the study was apparent, which was not because of having a recurrent stroke. However, the reason for 175 patients dropping out of the study was poor communication between the patient and provider, changes in contact information, and having a recurrent stroke may be one reason for not being able to make follow-up appointments. This may lead to biases in our results, as the patients lost to follow-up may have different outcomes than those who remained in the study. However, the sensitivity analysis showed no obvious bias when the lost follow-up patients (n=175) were excluded. Finally, in this study, the recurrent stroke was self-reported through follow-up interviews. Therefore, we reviewed the patient’s medical records to confirm the diagnosis of recurrent stroke, which requires magnetic resonance imaging or a computed tomography scan. If the patients could not provide their medical records, a family member or caretaker could provide information. Furthermore, having a recurrent stroke is likely related to being unable to make follow-up appointments and therefore dropping out of the study.

In this population-based cohort, despite being on oral anticoagulation following international guidelines, patients who suffer a stroke are at high risk of recurrent ischemic stroke. In addition, we found that a higher RDW at baseline was associated with an increased risk of recurrent ischemic stroke. An imbalance of oxidative stress and inflammatory response will increase the level of RDW. Based on the level of RDW, the clinician can identify the ischemic stroke patients who were appropriate for anti-inflammatory and antioxidant treatment. Therefore, routine clinical blood index (RDW) can provide a new perspective for managing recurrent ischemic stroke.

## MATERIALS AND METHODS

### Study design

The Ethics Committee of the Institute of Shanghai Xuhui Central Hospital, Shanghai, China, approved the parent study. Informed consent was obtained from all subjects. In this population-based retrospective cohort study performed from January 2007 to December 2017, acute ischemic stroke patients were prospectively collected from the Stroke center of Shanghai Xuhui Central Hospital, Fudan University. In this study, the diagnosis of ischemic stroke was according to the 2013 update of the American Heart Association/American Stroke Association’s definition of stroke [32].

### Study population

**Figure 3:**
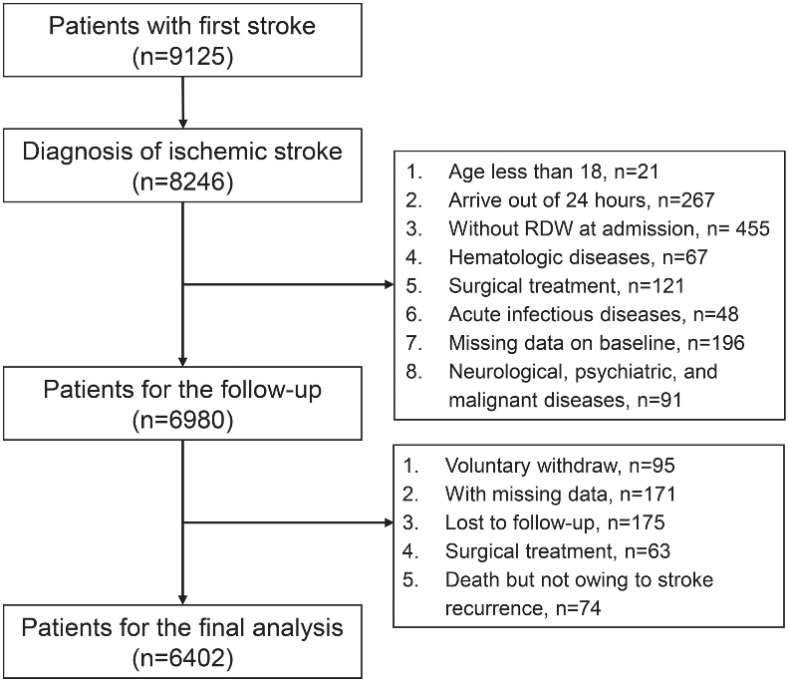
Flow chart of enrollment of participants in the study.

A total of 9125 patients with the first stroke were enrolled. Patients with a prior stroke before 2007 were excluded. Of these, 2145 patients were excluded based on inclusion and exclusion criteria, resulting in a sample size of 6980. A total of 578 patients were excluded from the study during the follow-up period, leaving 6402 subjects (Figure 3). Magnetic resonance imaging or computed tomography scan was required for all the patients to exclude intracranial hemorrhage. No patients with missing data were included in this study.

The inclusion criteria were: (1) age >18 years; (2) a diagnosis of ischemic stroke following the 2013 update of the American Heart Association/American Stroke Association’s definition of stroke [32]; (3) arrival within 24 h of stroke onset; (4) first ischemic stroke (large-artery atherosclerosis, cardioembolism, small-vessel occlusion, etc.); (5) having detailed clinical, laboratory, and follow-up data.

The exclusion criteria were as follows: (1) Patients with hemorrhagic stroke were excluded from the study, as this type of stroke is distinct from the ischemic stroke that was the focus of the study; (2) Patients who had undergone surgical treatment were excluded, as this could potentially influence the patient’s risk of recurrence; (3) Patients who had taken medications such as vasoactive drugs within one week of the study were excluded, as they could influence the RDW levels; (4) Patients with hematologic diseases and acute infectious diseases were excluded, as these conditions could also influence the RDW levels; (5) Patients with neurological, psychiatric, and malignant diseases, were excluded, as these conditions are related to not being able to make follow-up appointments; (6) Any patient with missing data on the baseline was excluded, as this could lead to an incomplete analysis.

### Outcome evaluation

After the first stroke, patients have interviewed annually face-to-face at three and twelve months after the stroke. The duration of follow-up was at least five years. Patient follow-ups started at the moment of the first ischemic stroke events. In this study, the recurrent stroke was self-reported through follow-up interviews. Survival status, cause of death, recurrence status, period of recurrence, therapy method, and medical records were recorded. We reviewed the patient’s medical records to confirm the diagnosis of recurrent stroke, which requires magnetic resonance imaging or a computed tomography scan. If the patients could not provide their medical records, a family member or caretaker could provide information. Following international guidelines, oral anticoagulation was initiated after the first ischemic stroke [33].

During the study, ischemic stroke recurrence was the primary outcome measure. Recurrent ischemic stroke has been defined as the sudden onset of a new focal neurological deficit caused by a vascular origin in the territory of a major cerebral artery [34].

### Laboratory assays

Laboratory examinations were performed at the Department of Clinical Laboratory, Shanghai Xuhui Central Hospital. Blood samples were collected within 24 h of admission. Blood samples (2 mL) for routine blood examination were collected in an EDTA anticoagulant tube. WBC, RBC, HGB, blood platelet count (PLT), and RDW were measured with the Sysmex series automated blood counting system (Kobe, Japan) within 0.5 h after blood collection.

In the meanwhile, measurements of aspartate aminotransferase (AST), alanine aminotransferase (ALT), gamma-glutamyl transpeptidase (GGT), BUN, CREA, and GLU were made using a 4 ml blood sample that was taken in the morning after the individuals had fasted for 8 hours. Within three hours of blood collection, the serum levels of AST, ALT, GGT, BUN, CREA, and GLU were determined by enzymatic colorimetry using a commercially available kit (Mindray, Shenzhen, China). Daily analysis of internal controls was conducted for ten years, and there were no significant changes in the coefficients of variation.

### Sample size calculation

We used an open-source calculator to calculate the minimum total sample size (http://www.powerandsamplesize.com/). Sample size calculation was based on a two-group comparison – the hazard ratio (HR): Cox PH, 2-Sided Equality. The HR is the ratio of the hazards between two groups. The input parameters were HR = 1.5, the overall probability of event = 0.04, proportion of the sample in group ‘A’=0.5, type I error rate = 0.05, power, 1-β=0.8. Based on this calculation, the minimum sample size required was 4765.

### Statistical analysis

Data were analyzed using SPSS 23.0 (SPSS, Chicago, IL, USA), and figures were rendered using GraphPad Prism 6. A Kolmogorov-Smirnov test was used to determine normality. Chi-square tests performed comparisons of categorical variables, and independent sample t-tests conducted comparisons of continuous variables. The relationship between pretreatment RDW levels and recurrent ischemic stroke HR was determined using a generalized additive model (GAM). We calculated HRs and 95% confidence intervals using Cox proportional hazard models. Kaplan-Meier curve was also used to calculate the clinical endpoints of the patients, and a log-rank test was used to compare them. P < 0.05 (two-sided) was considered statistical significance.

## Supplementary Material

Supplementary Table 1

## References

[1] GBD 2019 Stroke Collaborators. Global, regional, and national burden of stroke and its risk factors, 1990-2019: a systematic analysis for the Global Burden of Disease Study 2019. Lancet Neurol. 2021; 20:795–820. 10.1016/S1474-4422(21)00252-0 34487721 PMC8443449

[2] Donkor ES. Stroke in the 21^st^ Century: A Snapshot of the Burden, Epidemiology, and Quality of Life. Stroke Res Treat. 2018; 2018:3238165. 10.1155/2018/3238165 30598741 PMC6288566

[3] Ding Q, Liu S, Yao Y, Liu H, Cai T, Han L. Global, Regional, and National Burden of Ischemic Stroke, 1990-2019. Neurology. 2022; 98:e279–90. 10.1212/WNL.0000000000013115 34911748

[4] Perera KS, de Sa Boasquevisque D, Rao-Melacini P, Taylor A, Cheng A, Hankey GJ, Lee S, Fabregas JM, Ameriso SF, Field TS, Arauz A, Coutts SB, Arnold M, et al, and Young ESUS Investigators. Evaluating Rates of Recurrent Ischemic Stroke Among Young Adults With Embolic Stroke of Undetermined Source: The Young ESUS Longitudinal Cohort Study. JAMA Neurol. 2022; 79:450–8. 10.1001/jamaneurol.2022.0048 35285869 PMC8922202

[5] Vodencarevic A, Weingärtner M, Caro JJ, Ukalovic D, Zimmermann-Rittereiser M, Schwab S, Kolominsky-Rabas P. Prediction of Recurrent Ischemic Stroke Using Registry Data and Machine Learning Methods: The Erlangen Stroke Registry. Stroke. 2022; 53:2299–306. 10.1161/STROKEAHA.121.036557 35360927

[6] Zhang C, Wang Y, Zhao X, Liu L, Wang C, Pu Y, Zou X, Pan Y, Wong KS, Wang Y, and Chinese IntraCranial AtheroSclerosis (CICAS) Study Group. Prediction of Recurrent Stroke or Transient Ischemic Attack After Noncardiogenic Posterior Circulation Ischemic Stroke. Stroke. 2017; 48:1835–41. 10.1161/STROKEAHA.116.016285 28626054

[7] Sumi S, Origasa H, Houkin K, Terayama Y, Uchiyama S, Daida H, Shigematsu H, Goto S, Tanaka K, Miyamoto S, Minematsu K, Matsumoto M, Okada Y, et al. A modified Essen stroke risk score for predicting recurrent cardiovascular events: development and validation. Int J Stroke. 2013; 8:251–7. 10.1111/j.1747-4949.2012.00841.x 22759563

[8] Diener HC, Ringleb PA, Savi P. Clopidogrel for the secondary prevention of stroke. Expert Opin Pharmacother. 2005; 6:755–64. 10.1517/14656566.6.5.755 15934902

[9] Xie KH, Liu LL, Liang YR, Su CY, Li H, Liu RN, Chen QQ, He JS, Ruan YK, He WK. Red cell distribution width: a novel predictive biomarker for stroke risk after transient ischaemic attack. Ann Med. 2022; 54:1167–77. 10.1080/07853890.2022.2059558 35471128 PMC9045760

[10] Feng GH, Li HP, Li QL, Fu Y, Huang RB. Red blood cell distribution width and ischaemic stroke. Stroke Vasc Neurol. 2017; 2:172–5. 10.1136/svn-2017-000071 28989807 PMC5628378

[11] Pinho J, Marques SA, Freitas E, Araújo J, Taveira M, Alves JN, Ferreira C. Red cell distribution width as a predictor of 1-year survival in ischemic stroke patients treated with intravenous thrombolysis. Thromb Res. 2018; 164:4–8. 10.1016/j.thromres.2018.02.002 29438871

[12] Lappegård J, Ellingsen TS, Skjelbakken T, Mathiesen EB, Njølstad I, Wilsgaard T, Brox J, Brækkan SK, Hansen JB. Red cell distribution width is associated with future risk of incident stroke. The Tromsø Study. Thromb Haemost. 2016; 115:126–34. 10.1160/TH15-03-0234 26290352

[13] Peng M, Chen Y, Chen Y, Feng K, Shen H, Huang H, Zhao W, Zou H, Ji J. The relationship between red blood cell distribution width at admission and post-stroke fatigue in the acute phase of acute ischemic stroke. Front Neurol. 2022; 13:922823. 10.3389/fneur.2022.922823 35968310 PMC9366669

[14] Fan H, Liu X, Li S, Liu P, Song Y, Wang H, Tang X, Luo Y, Li J, Zhu Y, Chen Y. High red blood cell distribution width levels could increase the risk of hemorrhagic transformation after intravenous thrombolysis in acute ischemic stroke patients. Aging (Albany NY). 2021; 13:20762–73. 10.18632/aging.203465 34449439 PMC8436933

[15] Smock KJ, Perkins SL. Thrombocytopenia: an update. Int J Lab Hematol. 2014; 36:269–78. 10.1111/ijlh.12214 24750673

[16] Rost NS, Giugliano RP, Ruff CT, Murphy SA, Crompton AE, Norden AD, Silverman S, Singhal AB, Nicolau JC, SomaRaju B, Mercuri MF, Antman EM, Braunwald E, and ENGAGE AF-TIMI 48 Investigators. Outcomes With Edoxaban Versus Warfarin in Patients With Previous Cerebrovascular Events: Findings From ENGAGE AF-TIMI 48 (Effective Anticoagulation With Factor Xa Next Generation in Atrial Fibrillation-Thrombolysis in Myocardial Infarction 48). Stroke. 2016; 47:2075–82. 10.1161/STROKEAHA.116.013540 27387994

[17] Diener HC, Connolly SJ, Ezekowitz MD, Wallentin L, Reilly PA, Yang S, Xavier D, Di Pasquale G, Yusuf S, and RE-LY study group. Dabigatran compared with warfarin in patients with atrial fibrillation and previous transient ischaemic attack or stroke: a subgroup analysis of the RE-LY trial. Lancet Neurol. 2010; 9:1157–63. 10.1016/S1474-4422(10)70274-X 21059484

[18] Hankey GJ, Patel MR, Stevens SR, Becker RC, Breithardt G, Carolei A, Diener HC, Donnan GA, Halperin JL, Mahaffey KW, Mas JL, Massaro A, Norrving B, et al, and ROCKET AF Steering Committee Investigators. Rivaroxaban compared with warfarin in patients with atrial fibrillation and previous stroke or transient ischaemic attack: a subgroup analysis of ROCKET AF. Lancet Neurol. 2012; 11:315–22. 10.1016/S1474-4422(12)70042-X 22402056

[19] Song SY, Hua C, Dornbors D 3rd, Kang RJ, Zhao XX, Du X, He W, Ding YC, Meng R. Baseline Red Blood Cell Distribution Width as a Predictor of Stroke Occurrence and Outcome: A Comprehensive Meta-Analysis of 31 Studies. Front Neurol. 2019; 10:1237. 10.3389/fneur.2019.01237 31849813 PMC6901990

[20] Turcato G, Cappellari M, Follador L, Dilda A, Bonora A, Zannoni M, Bovo C, Ricci G, Bovi P, Lippi G. Red Blood Cell Distribution Width Is an Independent Predictor of Outcome in Patients Undergoing Thrombolysis for Ischemic Stroke. Semin Thromb Hemost. 2017; 43:30–5. 10.1055/s-0036-1592165 27813042

[21] He M, Wang H, Tang Y, Cui B, Xu B, Niu X, Sun Y, Zhang G, He X, Wang B, Xu B, Li Z, Zhang Y, Wang Y. Red blood cell distribution width in different time-points of peripheral thrombolysis period in acute ischemic stroke is associated with prognosis. Aging (Albany NY). 2022; 14:5749–67. 10.18632/aging.204174 35832033 PMC9365566

[22] Xue J, Zhang D, Zhang XG, Zhu XQ, Xu XS, Yue YH. Red cell distribution width is associated with stroke severity and unfavorable functional outcomes in ischemic stroke. Front Neurol. 2022; 13:938515. 10.3389/fneur.2022.938515 36438973 PMC9682065

[23] Hong RH, Zhu J, Li ZZ, Yuan J, Zhao P, Ding J, Fan QL, Yang J, Liu BG, Cai J, Zhu DS, Guan YT. Red blood cell distribution width is associated with neuronal damage in acute ischemic stroke. Aging (Albany NY). 2020; 12:9855–67. 10.18632/aging.103250 32445553 PMC7288978

[24] Kara H, Degirmenci S, Bayir A, Ak A, Akinci M, Dogru A, Akyurek F, Kayis SA. Red cell distribution width and neurological scoring systems in acute stroke patients. Neuropsychiatr Dis Treat. 2015; 11:733–9. 10.2147/NDT.S81525 25834448 PMC4370912

[25] Lippi G, Targher G, Montagnana M, Salvagno GL, Zoppini G, Guidi GC. Relation between red blood cell distribution width and inflammatory biomarkers in a large cohort of unselected outpatients. Arch Pathol Lab Med. 2009; 133:628–32. 10.5858/133.4.628 19391664

[26] Semba RD, Patel KV, Ferrucci L, Sun K, Roy CN, Guralnik JM, Fried LP. Serum antioxidants and inflammation predict red cell distribution width in older women: the Women’s Health and Aging Study I. Clin Nutr. 2010; 29:600–4. 10.1016/j.clnu.2010.03.001 20334961 PMC3243048

[27] Kiefer CR, Snyder LM. Oxidation and erythrocyte senescence. Curr Opin Hematol. 2000; 7:113–6. 10.1097/00062752-200003000-00007 10698298

[28] He J, Liu J, Huang Y, Tang X, Xiao H, Hu Z. Oxidative Stress, Inflammation, and Autophagy: Potential Targets of Mesenchymal Stem Cells-Based Therapies in Ischemic Stroke. Front Neurosci. 2021; 15:641157. 10.3389/fnins.2021.641157 33716657 PMC7952613

[29] Kersten CJB, Zandbergen AAM, Fokkert MJ, Slingerland RJ, den Hertog HM. Continuous glucose monitoring in acute ischemic stroke patients treated with endovascular therapy: A pilot study to assess feasibility and accuracy. PLoS One. 2023; 18:e0280153. 10.1371/journal.pone.0280153 36758045 PMC9910721

[30] Osei E, Fonville S, Zandbergen AAM, Koudstaal PJ, Dippel DWJ, den Hertog HM. Impaired fasting glucose is associated with unfavorable outcome in ischemic stroke patients treated with intravenous alteplase. J Neurol. 2018; 265:1426–31. 10.1007/s00415-018-8866-z 29666986

[31] Peng R, Liu K, Li W, Yuan Y, Niu R, Zhou L, Xiao Y, Gao H, Yang H, Zhang C, Zhang X, He M, Wu T. Blood urea nitrogen, blood urea nitrogen to creatinine ratio and incident stroke: The Dongfeng-Tongji cohort. Atherosclerosis. 2021; 333:1–8. 10.1016/j.atherosclerosis.2021.08.011 34390959

[32] Sacco RL, Kasner SE, Broderick JP, Caplan LR, Connors JJB, Culebras A, Elkind MS, George MG, Hamdan AD, Higashida RT, Hoh BL, Janis LS, Kase CS, et al, and American Heart Association Stroke Council, Council on Cardiovascular Surgery and Anesthesia, and Council on Cardiovascular Radiology and Intervention, and Council on Cardiovascular and Stroke Nursing, and Council on Epidemiology and Prevention, and Council on Peripheral Vascular Disease, and Council on Nutrition, Physical Activity and Metabolism. An updated definition of stroke for the 21st century: a statement for healthcare professionals from the American Heart Association/American Stroke Association. Stroke. 2013; 44:2064–89. 10.1161/STR.0b013e318296aeca 23652265 PMC11078537

[33] Klijn CJ, Paciaroni M, Berge E, Korompoki E, Kõrv J, Lal A, Putaala J, Werring DJ. Antithrombotic treatment for secondary prevention of stroke and other thromboembolic events in patients with stroke or transient ischemic attack and non-valvular atrial fibrillation: A European Stroke Organisation guideline. Eur Stroke J. 2019; 4:198–223. 10.1177/2396987319841187 31984228 PMC6960695

[34] Paciaroni M, Caso V, Agnelli G, Mosconi MG, Giustozzi M, Seiffge DJ, Engelter ST, Lyrer P, Polymeris AA, Kriemler L, Zietz A, Putaala J, Strbian D, et al. Recurrent Ischemic Stroke and Bleeding in Patients With Atrial Fibrillation Who Suffered an Acute Stroke While on Treatment With Nonvitamin K Antagonist Oral Anticoagulants: The RENO-EXTEND Study. Stroke. 2022; 53:2620–7. 10.1161/STROKEAHA.121.038239 35543133

