# Supplementary material for: Association between red blood cell distribution width and ischemic stroke recurrence in patients with acute ischemic stroke: a 10-years retrospective cohort analysis
Source: Aging (Albany NY). 2023 Apr 12;15(8):3052–63. doi: 10.18632/aging.204657 (PMC10188350; doi:10.18632/aging.204657)
Supplement: Supplementary Table 1 [file aging-15-204657-s001.pdf]

## SUPPLEMENTARY TABLE

**Supplementary Table 1. Sensitivity analysis: association of baseline RDW levels with recurrent ischemic stroke.**

| | Crude model | | Adjusted model* | |
|---|---|---|---|---|
| | **HR (95%CI)** | **P value** | **HR (95%CI)** | **P value** |
| Model A | | | | |
| Quartile | | | | |
| Q1 (<12.4) | Ref | | Ref | |
| Q2 (12.4 to 12.8) | 1.003 (0.937-1.073) | 0.932 | 1.003 (0.936-1.075) | 0.927 |
| Q3 (12.8 to 13.3) | 1.169 (1.069-1.285) | 0.076 | 1.166 (1.066-1.286) | 0.050 |
| Q4 (>13.3) | 1.222 (1.091-1.369) | 0.001 | 1.211 (1.075-1.364) | 0.002 |
| P for trend | | <0.001 | | <0.001 |
| Categories | | | | |
| Q1-Q2 (<12.8) | Ref | | Ref | |
| Q3-Q4 (>12.8) | 1.305 (1.142-1.491) | <0.001 | 1.313 (1.144-1.506) | <0.001 |
| Model B | | | | |
| Quartile | | | | |
| Q1 (<12.4) | Ref | | Ref | |
| Q2 (12.4 to 12.8) | 1.003 (0.938-1.073) | 0.921 | 1.003 (0.937-1.074) | 0.932 |
| Q3 (12.8 to 13.3) | 1.225 (1.006-1.505) | 0.044 | 1.248 (1.011-1.541) | 0.039 |
| Q4 (>13.3) | 1.289 (1.130-1.470) | <0.001 | 1.293 (1.127-1.485) | <0.001 |
| P for trend | | <0.001 | | <0.001 |
| Categories | | | | |
| Q1-Q2 (<12.8) | Ref | | Ref | |
| Q3-Q4 (>12.8) | 1.548 (1.319-1.818) | <0.001 | 1.581 (1.340-1.864) | <0.001 |

*Adjusted for age, sex, hypertension, diabetes mellitus, smoking, drinking, body mass index, and coronary heart disease. Q, quartile. Model A: all the lost to follow up patients (n=175) were considered as stroke recurrence. Model B: all the lost to follow up patients (n=175) were considered as non-stroke recurrence.
